# Improving the alkali metal electrode/inorganic solid electrolyte contact via room-temperature ultrasound solid welding

**DOI:** 10.1038/s41467-021-27473-4

**Published:** 2021-12-07

**Authors:** Xinxin Wang, Jingjing Chen, Dajian Wang, Zhiyong Mao

**Affiliations:** 1 Tianjin Key Laboratory for Photoelectric Materials and Devices, School of Materials Science and Engineering, Tianjin University of Technology, Tianjin, 300384 P. R. China; 2 Key Laboratory of Display Materials and Photoelectric Devices, Tianjin University of Technology, Ministry of Education, Tianjin, 300384 P. R. China

**Keywords:** Batteries, Batteries, Nanoscale materials, Energy

## Abstract

The combination of alkali metal electrodes and solid-state electrolytes is considered a promising strategy to develop high-energy rechargeable batteries. However, the practical applications of these two components are hindered by the large interfacial resistance and growth of detrimental alkali metal depositions (e.g., dendrites) during cycling originated by the unsatisfactory electrode/solid electrolyte contact. To tackle these issues, we propose a room temperature ultrasound solid welding strategy to improve the contact between Na metal and Na_3_Zr_2_Si_2_PO_12_ (NZSP) inorganic solid electrolyte. Symmetrical Na|NZSP | Na cells assembled via ultrasonic welding show stable Na plating/stripping behavior at a current density of 0.2 mA cm^−2^ and a higher critical current density (i.e., 0.6 mA cm^−2^) and lower interfacial impedance than the symmetric cells assembled without the ultrasonic welding strategy. The beneficial effect of the ultrasound welding is also demonstrated in Na|NZSP | Na_3_V_2_(PO_4_)_3_ full coin cell configuration where 900 cycles at 0.1 mA cm^−2^ with a capacity retention of almost 90% can be achieved at room temperature.

## Abstract (web summary)

The practical application of solid-state rechargeable alkali metal batteries is hindered by the poor contact between the metal electrode and the solid electrolyte. Here, the authors report an ultrasound solid welding strategy to favor the contact between these cells components.

## Introduction

Commercial lithium ions batteries (LIBs) have promoted the rapid development of electrochemical energy storage technology in the past decades. However, the energy density of LIBs reached a bottleneck and the safety issues of liquid organic electrolyte used in LIBs were denounced^1,2^. Solid-state alkali metal batteries employing solid-state electrolytes are recognized as the next-generation energy storage technology due to their merits in high-energy density and safety performances as well as long-life time^3,4^. Nevertheless, the practical application of solid-state batteries is hindered seriously by the poor contact between electrodes and solid-state electrolyte as well as the resultant interfacial issues^5^. Such as, the poor contact of interface leads to a huge interface resistance^6,7^, preventing the effective transport of ions; the uneven deposition/stripping of metal anode at the discontinuous contact interface results in the growth of metal dendrites during the electrochemical cycling process, eventually causing the failure of batteries^8,9^. Among various types of solid-state electrolytes, oxide ceramic electrolytes have been closely concerned in view of their high ion conductivity (10^−4 ^~ 10^−3^ S cm^−1^) and wide voltage window^10,11^. Especially, the good mechanical strength of oxide ceramic electrolytes can effectively alleviate and inhibit the dendritic growth of alkali metal anode^12,13^. However, the problem of poor contact between metal anode and rigid oxide ceramic electrolytes is most prominent among the solid-state batteries involving in various types of solid-state electrolytes.

A series of strategies have been approached to improve the interface contact between metal electrodes and oxide ceramic electrolytes^14,15^. Static pressing was mostly employed to improve the physical contact between the metal anode and solid-state electrolyte to a certain extent, but the improvement effect of interface contact with acceptable interface impedance is limited^16^. Fusion welding with the assistance of heating alkali metals beyond their melting points (sodium: 98 ^o^C, lithium: 180 ^o^C) was widely used to improve the interface contact of metals anode with solid electrolyte^17^. However, the wettability of molten metal to oxide ceramic was hindered seriously because of the formation of undesired layer on the surface of ceramic electrolyte^18–20^. Very recently, ultrasonication was employed to aggressively promote the joining of molten Li with Li_7_La_3_Zr_2_O_12_ (LLZO) and this technology was referred as ultrasonic-assisted fusion welding method^21^. No matter the traditional fusion welding or the newly exploited ultrasonic-assisted fusion welding, heating operation for the formation of molten metals is required. Apparently, the heating operation will complicate the assemble of batteries and is unsuitable for large-scale application. The introduction of an interlayer with lithiophilic /sodiophilic character, exampling with ZnO^22^, Al_2_O_3_^5^, Li_3_PO_4_^23^, TiO_2_^24^, Li_3_N^25^, and others, between alkali metal anode and ceramic electrolyte was recognized as an effective route to promote the interface contact, resulting in the obvious decrease of the interface resistance and improved uniform deposition of the alkali metal. For example, Lu et al. fabricated a 3D porous structure Na_3_Zr_2_Si_2_PO_12_ (NZSP) impregnated with SnO_2_ on its surface and then filled with molten sodium, and reported the application performances in solid-state sodium metal battery^26^. Miao et al. reported that the AlF_3_ interlayer between the sodium anode and NZSP electrolyte could induce the uniform deposition and stripping of Na^27^. Nevertheless, the interlayer with poor adhesion might partially peel off and lead to the interface contact becomes even worse, accompanying with the volume change of electrode and the complex electrochemical process during cycling. Thus, it is urgent to develop promising interface joining technology to fabricate intimate contact interface with atom-level bonding between metal anodes and ceramic electrolytes.

The application of ultrasonic welding has been successfully demonstrated in joining metal and ceramic in the past years^28–31^. High-frequency ultrasonic vibration energy could be converted into the deformation energy, frictional work and limited temperature rise, leading to the quick diffusion of atoms at the interface and the formation of intimate bonding interface^29^. Especially for the joining of metal and ceramic, ultrasonic friction could quickly break oxide film on metal surface and gas film on ceramic surface, promoting the wettability spreading of metal to ceramic^32–34^.

In this work, room temperature ultrasonic solid welding without heating operation is demonstrated to fabricate an intimate contact interface between sodium metal anode and Na_3_Zr_2_Si_2_PO_12_ ceramic electrolyte. The resultant interface properties and the boosting application performances in solid-state sodium metal batteries are investigated in detail.

## Results

### Characterizations of Na_3_Zr_2_Si_2_PO_12_ electrolyte

**Fig. 1:**
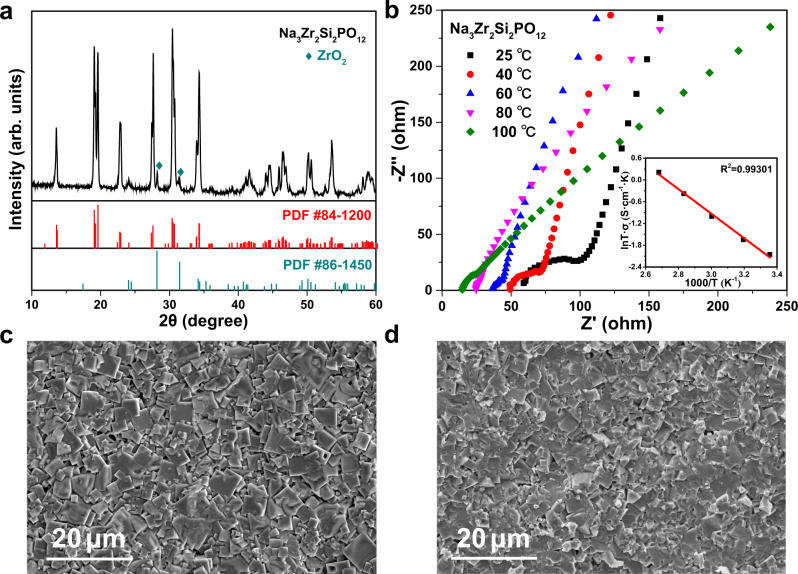
Physicochemical characterizations of the Na_3_Zr_2_Si_2_PO_12_ solid electrolyte. **a** XRD diffraction patterns. **b** Nyquist plots and Arrhenius plots of impedance. **c** Top SEM view. **d** SEM image of the cross-section.

Figure 1a depicted the XRD pattern of the prepared Na_3_Zr_2_Si_2_PO_12_ ceramic pellets, which could be well indexed as the NASICON-type Na_3_Zr_2_Si_2_PO_12_ targeted phase (PDF #84–1200). Two weak peaks positioned at ~28.28° and ~31.51° were corresponded to the impurity phase of ZrO_2_ (PDF #86–1450). The coexistence of small amount of ZrO_2_ impurity was a common phenomenon caused by the volatility of Na and P during high temperature calcination^35–38^. The Nyquist plots of impedance for the prepared Na_3_Zr_2_Si_2_PO_12_ sheet at different temperature were shown in Fig. 1b. The impedance spectra of electrolyte consisted of a semicircle at high frequency and a diagonal line at low frequency. The size of semicircle represented the grain boundary resistance, and the intercept of semicircle with *X*-axis was the total resistance of bulk and grain boundary. The ionic conductivity of the obtained electrolyte at room temperature was calculated to be 4.3 × 10^−4^ S cm^−1^. With the increasing of test temperature, the semicircle representing the grain boundary resistance decreased gradually, indicating that the grain boundary resistance occupied a smaller proportion of the total resistance at high temperature. The activation energy (E_a_) was determined to be 0.291 eV from the slope of the Arrhenius plot in the form of ln (σT) versus 1000/T. The top SEM view of the prepared electrolyte was shown in Fig. 1c. It was shown that the crystalline grains of Na_3_Zr_2_Si_2_PO_12_ show a cube-like morphology and some gaps existed between grains. In addition, the cross-section SEM image (Fig. 1d) of Na_3_Zr_2_Si_2_PO_12_ pellet showed that there were voids in the ceramic even though the electrolyte has the acceptable ionic conductivity (4.3 × 10^−4^ S cm^−1^ at room temperature) and high density (the density of the prepared electrolyte pellets was 3.10 ~ 3.13 g cm^−3^ with a relative density of ~95%).

### Fabrication of intimate contact interface by ultrasound welding

**Fig. 2:**
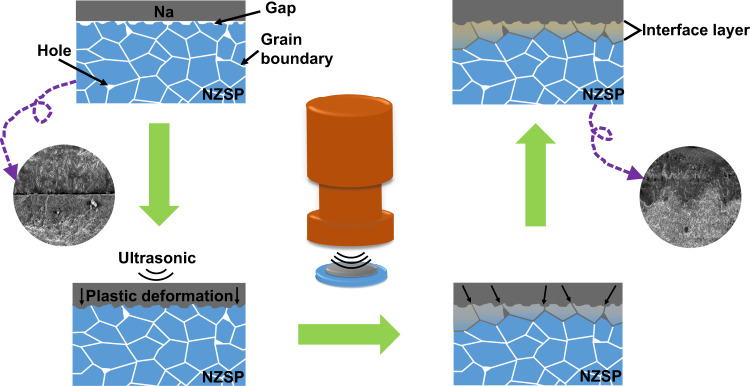
Schematic illustration of the ultrasound solid welding method. Ultrasonic welding improved the metal Na/NZSP solid electrolyte from poor contact to continuous close contact, and formed a stable interface layer.

Room temperature ultrasound solid welding was employed to fabricate the intimate contact interface between Na metal anodes and Na_3_Zr_2_Si_2_PO_12_ (NZSP) electrolytes, as schematically illustrated in Fig. 2. The Na metal foil was placed on the top of Na_3_Zr_2_Si_2_PO_12_ pellet, then the ultrasonic horn of a household ultrasonic cleaner (photographic picture, power and frequency of the household ultrasonic cleaner was shown in Supplementary Fig. 1) was loaded on the Na metal foil with ultrasonic vibration for ~25 s at room temperature (25–27 °C) (Supplementary Movie). The fabricated intimate interface between Na anode and Na_3_Zr_2_Si_2_PO_12_ electrolyte by ultrasound welding was indexed as UW-Na/Na_3_Zr_2_Si_2_PO_12_. As a comparison, Na/Na_3_Zr_2_Si_2_PO_12_ interface without ultrasonic vibration was fabricated by static pressing method (Kejing MSK-160E, China) with a pressure intensity of ~300 kg (~26 MPa). Supplementary Fig. 2a displayed the digital photographs of the Na/Na_3_Zr_2_Si_2_PO_12_, in which Na metal anode fabricated by static pressing could be scraped off easily from the surface of Na_3_Zr_2_Si_2_PO_12_ pellet, revealing the contact between Na and electrolyte pellet is poor. On the contrary, Na electrode still had a large area residue on the surface of Na_3_Zr_2_Si_2_PO_12_ pellet for the UW-Na/Na_3_Zr_2_Si_2_PO_12_ even after scraping with a knife, indicating the intimate contact between Na foil and Na_3_Zr_2_Si_2_PO_12_ pellet with the aid of ultrasonic welding (Supplementary Movie and Supplementary Fig. 2b).

### Characterizations of interface properties

**Fig. 3:**
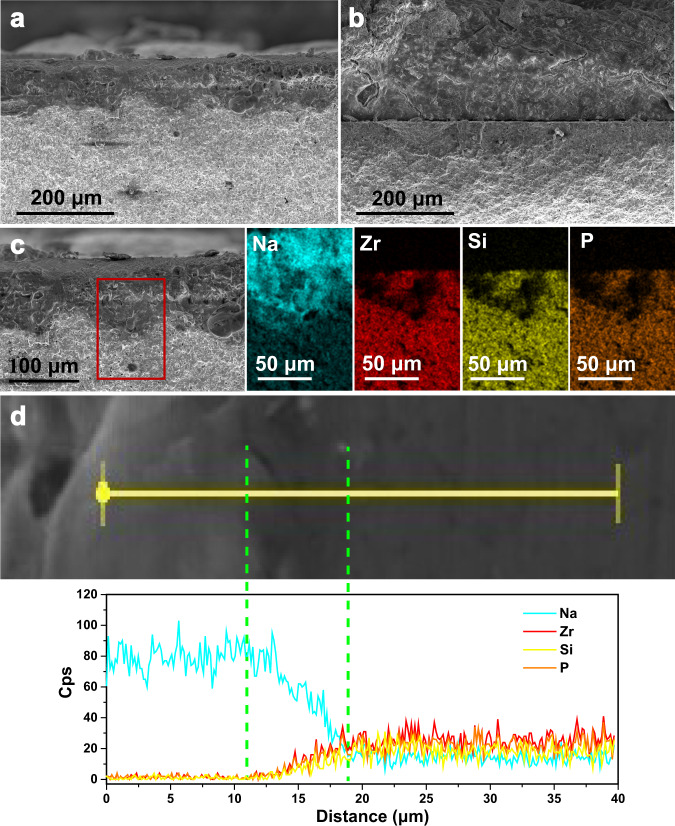
Ex situ morphological and chemical characterizations of the metal electrode/solid electrolyte interface. **a** SEM image of the cross-section of UW-Na/Na_3_Zr_2_Si_2_PO_12_. **b** SEM image of the cross-section of Na/Na_3_Zr_2_Si_2_PO_12_ interfaces. **c** EDX elemental mappings for UW-Na/Na_3_Zr_2_Si_2_PO_12_. **d** EDX elemental line scan profile for UW-Na/Na_3_Zr_2_Si_2_PO_12_.

The contact property of the interface between Na metal and Na_3_Zr_2_Si_2_PO_12_ pellet was evaluated by the cross-sectional scanning electron microscopy (SEM) image, as shown in Fig. 3a. The formed interface between Na metal and Na_3_Zr_2_Si_2_PO_12_ pellet for UW-Na/Na_3_Zr_2_Si_2_PO_12_ was intimate contact without microscopic void defects. In contrast, the Na/Na_3_Zr_2_Si_2_PO_12_ interface (Fig. 3b) constructed by static pressing without ultrasound welding exhibited obviously gap between Na anode and Na_3_Zr_2_Si_2_PO_12_ electrolyte, resulting in large interfacial resistance because of the poor ions transport at the interface^39^. This result verified that ultrasound solid welding was an effective method to construct intimate contact interface between Na anode and Na_3_Zr_2_Si_2_PO_12_ electrolyte. The EDX elemental mapping of the selected cross-sectional area for UW-Na/Na_3_Zr_2_Si_2_PO_12_ interface was reported in Fig. 3c. It was found that the elements of Zr, Si and P pertaining to Na_3_Zr_2_Si_2_PO_12_ pellet showed a similar distribution whereas the Na element has a significant diffusion from the Na anode into the surface thin layer of Na_3_Zr_2_Si_2_PO_12_ pellet. This revealed that the Na metal could diffuse into Na_3_Zr_2_Si_2_PO_12_ pellet along the grain boundaries or the pores of ceramics in view of its rapid plastic deformation under ultrasonic vibration^40,41^. Different from the sodium dendrites that grow along the grain boundary resulted from the poor contact between anode and solid electrolyte, intimate bonding interface with a stable interfacial layer was constructed through interfacial reaction between the diffused Na metal and Na_3_Zr_2_Si_2_PO_12_ with the aid of ultrasound. The interfacial reaction mechanism between the Na and Na_3_Zr_2_Si_2_PO_12_ induced by ultrasonication was revealed by the XRD data, as disclosed in Supplementary Fig. 3. Comparing with the pristine Na_3_Zr_2_Si_2_PO_12_ powder, the ultrasound welding sample of Na_3_Zr_2_Si_2_PO_12_ powder with sodium metal exhibits some new diffraction peaks: some are originated from the metal sodium but disappears once exposed in air, and others are indexed to be a newly-formed phase of Na_2_SiO_3_, revealing the formation of Na_2_SiO_3_ due to the interfacial reaction. Zhang et al. investigated that Na_3_Zr_2_Si_2_PO_12_ could be reduced by metal Na to produce Na_4_SiO_4_ and other reduction products at 0 V, and will further to produce Na_2_SiO_3_ at a higher voltages^42^. In the present work, the newly-formed Na_2_SiO_3_ product was detected during the interfacial reaction under ultrasonication. It was reported that Na_2_SiO_3_ was an ion conductor^43^ and 5 wt.% Na_2_SiO_3_ presented in Na_3_Zr_2_Si_2_PO_12_ might result in a high total ionic conductivity of 1.45 mS cm^−1^ ^44^, indicating that the formation of Na_2_SiO_3_ induced by ultrasonication is beneficial to promote the ions transport in the UW-Na/Na_3_Zr_2_Si_2_PO_12_ interface. The EDX elemental line scan was further used to confirm the successful formation of interface layer for UW-Na/Na_3_Zr_2_Si_2_PO_12_, as depicted in Fig. 3d. An interfacial reaction region with concentration gradient for the involving Na, Zr, Si and P elements was observed. To illustrate the generality of the proposed ultrasound solid welding in solid-state metal batteries, intimate contact interfaces between Li/Na metal and diversified ceramic electrolytes constructed by ultrasonication were demonstrated in Supplementary Figs. 4–8. The SEM image of the cross-section and the corresponding EDX elemental mappings for Li/Na_3_Zr_2_Si_2_PO_12_ (Supplementary Fig. 4), Na/Li_7_La_3_Zr_2_O_12_ (Supplementary Fig. 5), Li/Li_7_La_3_Zr_2_O_12_ (Supplementary Fig. 6), Na/Na-β''-Al_2_O_3_ (Supplementary Fig. 7) and Li/Na-β''-Al_2_O_3_ (Supplementary Fig. 8) interfaces revealed that these interfaces constructed by ultrasonication are intimate without microscopic void defects. Above results verified that ultrasound solid welding is an effective route to construct intimate contact interface between alkali metals anode and ceramic electrolytes, beneficiating to settle the interfacial issues denounced in solid-state alkali metals batteries.

### Testing of solid-state cells assembled via ultrasonic welding method

**Fig. 4:**
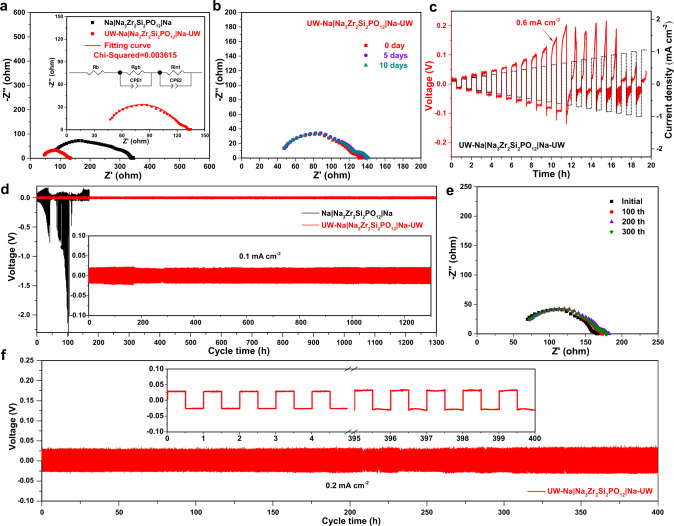
Electrochemical characterizations of symmetrical Na metal coin cells with Na_3_Zr_2_Si_2_PO_12_ solid electrolyte. **a** The electrochemical impedance spectra for the assembled Na | Na_3_Zr_2_Si_2_PO_12_ | Na and UW-Na|Na_3_Zr_2_Si_2_PO_12_ | Na-UW symmetrical cells. **b** Electrochemical impedance spectra during the storage for 10 days of the assembled UW-Na|Na_3_Zr_2_Si_2_PO_12_ | Na-UW symmetrical cell. **c** Galvanostatic critical current density test. **d** Galvanostatic cycling of Na | Na_3_Zr_2_Si_2_PO_12_ | Na and UW-Na | Na_3_Zr_2_Si_2_PO_12_ | Na-UW symmetrical cells at current density of 0.1 mA cm^−2^. **e** The electrochemical impedance spectra for the initial and cycled UW-Na | Na_3_Zr_2_Si_2_PO_12_ | Na-UW symmetrical cell at current density of 0.1 mA cm^−2^. **f** Galvanostatic cycling of UW-Na | Na_3_Zr_2_Si_2_PO_12_ | Na-UW symmetrical cell at current density of 0.2 mA cm^−2^.

The electrochemical impedance spectra of assembled Na | Na_3_Zr_2_Si_2_PO_12_ | Na and UW-Na | Na_3_Zr_2_Si_2_PO_12_ | Na-UW symmetrical cells were measured to evaluate their interface resistance, as displayed in Fig. 4a. Inset showed the fitted data and corresponding equivalent circuit, where R_b_, R_gb_, R_int_ and CPE indicated the bulk resistance, grain boundary resistance, interface resistance and constant phase element, respectively. The left intercept of the arc with *x*-axis at high frequency represents the bulk resistance of Na_3_Zr_2_Si_2_PO_12_ electrolyte whereas the arc represents to the overlap of grain boundary impedance and interface impedance^5^. The sum of R_b_ and R_gb_ could be designated as the total resistance of Na_3_Zr_2_Si_2_PO_12_ electrolyte, which was ~100 Ω measured in the Fig. 1b. R_int_ was determined for the interface resistance on either side of the Na symmetric cells by subtracting Na_3_Zr_2_Si_2_PO_12_ electrolyte resistance from UW-Na | Na_3_Zr_2_Si_2_PO_12_ | Na-UW or Na|Na_3_Zr_2_Si_2_PO_12_ | Na cell resistance, dividing by two, and then normalizing to the Na electrode surface area. The UW-Na | Na_3_Zr_2_Si_2_PO_12_ | Na-UW cell showed a significantly lower total resistance of ~140 Ω than the Na | Na_3_Zr_2_Si_2_PO_12_ | Na cell (~350 Ω) at room temperature. The interfacial resistance of UW-Na/Na_3_Zr_2_Si_2_PO_12_ was evaluated to be ~22.6 Ω cm^2^, which was lower than that of Na/Na_3_Zr_2_Si_2_PO_12_ (141.3 Ω cm^2^). This significantly lower interfacial resistance for UW-Na/Na_3_Zr_2_Si_2_PO_12_ was dominantly ascribed to the intimate contact and the formed ion conductive Na_2_SiO_3_ induced by ultrasonication. The evolution of electrochemical impedance spectra for the assembled UW-Na | Na_3_Zr_2_Si_2_PO_12_ | Na-UW symmetrical cell during the storage for 10 days was recorded in Fig. 4b. There was nearly no change for the impedance spectra with a constant interfacial resistance, indicating the constructed interface by ultrasonication was extremely stable. Galvanostatic critical current density (CCD) was a benchmark to evaluate the electroplating and stripping ability of Na at the interface under gradually increasing current density^45^. The CCD measurement result for the UW-Na | Na_3_Zr_2_Si_2_PO_12_ | Na-UW symmetrical cell was presented in Fig. 4c. It was observed that the maximum current density was recorded to be as high as 0.6 mA cm^−2^ with a capacity of 0.6 mA h cm^−2^, which was much higher than that of the most reported values (0.07~0.2 mA cm^−2^) for the Na | Na_3_Zr_2_Si_2_PO_12_ | Na symmetric cells without any interfacial modification^27,42,45^. Supplementary Fig. 9 displayed the EIS measurements of the correlated total electrochemical resistance during CCD measurements. When the current density was 0.1~0.35 mA cm^−2^, the recorded impedance spectra was changeless, implying the stability of the Na/Na_3_Zr_2_Si_2_PO_12_ interface. With the increase of current density to 0.4~0.6 mA cm^−2^, the impedance values of the battery were larger than the initial, implying the effective contact of UW-Na/Na_3_Zr_2_Si_2_PO_12_ interface was deteriorated due to Na dissolution and deposition at higher current density. The cycle stability of the assembled cells was evaluated by the Galvanostatic cycling measurements at a current density of 0.1 mA cm^−2^, as demonstrated in Fig. 4d. The UW-Na | Na_3_Zr_2_Si_2_PO_12_ | Na-UW symmetrical cell exhibited a stable voltage profile with a small overvoltage and without any voltage disturbance during a long cycling time of 1300 h. In contrast, the Na | Na_3_Zr_2_Si_2_PO_12_ | Na symmetric cell have a large voltage and short-circuited after only 30 h of cycling, resulting from the poor contact between Na and Na_3_Zr_2_Si_2_PO_12_. The nearly unchanged electrochemical impedance spectra (Fig. 4e) for the initial and cycled UW-Na | Na_3_Zr_2_Si_2_PO_12_ | Na-UW symmetrical cell also verified the cycling stability of the ultrasound welding constructed interface. At a higher current density of 0.2 mA cm^−2^, it was also found that the assembled UW-Na | Na_3_Zr_2_Si_2_PO_12_ | Na-UW symmetrical cell could be cycled firmly with stable voltage and changeless impedance values for a long cycling time (Fig. 4f and Supplementary Fig. 10). Supplementary Fig. 11a exhibited the cross-section of UW-Na/Na_3_Zr_2_Si_2_PO_12_ recovered from cycled Na symmetrical cells at 0.2 mA cm^−2^ after 100 h cycling. It was observed that the intimate contact interface between Na and Na_3_Zr_2_Si_2_PO_12_ could be maintained after cycling. Additionally, the relatively intact cube-like morphology of the electrolyte pellet surface after removing Na foil with ethanol also confirmed the stability of the UW-Na/Na_3_Zr_2_Si_2_PO_12_ interface (Supplementary Fig. 11b).

Supplementary Table 1 provided a detail comparison about the interface resistance and the cycling stability for Na | Na_3_Zr_2_Si_2_PO_12_ | Na symmetric cells improved with different interface modification routes. It was found that ultrasound solid welding technology facilitates the decrease of interface resistance and improvement of cycling stability comparing with the referring routes, enabling its promising application to settle the interfacial issues of solid-state metal batteries. These results confirmed that the fabricated intimate contact interface induced by ultrasonication not only significantly reduce the interface impedance but also build a stable interface layer to induce the uniform deposition and stripping of Na.

**Fig. 5:**
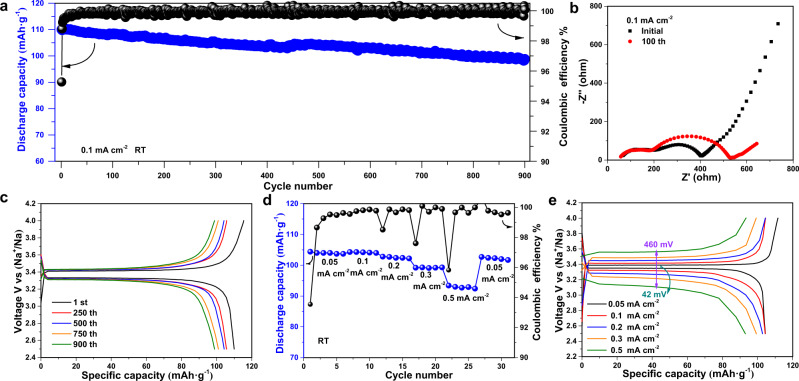
Electrochemical characterizations of full Na metal coin cells with Na_3_Zr_2_Si_2_PO_12_ solid electrolyte. **a** Cycling performance, **b** electrochemical impedance spectra and **c** typical charge/discharge curves of the assembled UW-Na | Na_3_Zr_2_Si_2_PO_12_ | Na_3_V_2_(PO_4_)_3_ solid-state sodium metal cell at 0.1 mA cm^−2^. **d** The rate performance and **e** typical charge/discharge curves of the assembled UW-Na | Na_3_Zr_2_Si_2_PO_12_ | Na_3_V_2_(PO_4_)_3_ cell at various current densities.

The cycling performance of the assembled UW-Na | Na_3_Zr_2_Si_2_PO_12_ | Na_3_V_2_(PO_4_)_3_ sodium metal battery at 0.1 mA cm^−2^ was displayed in Fig. 5a. The first discharge specific capacity was recorded to be 110 mAh·g^−1^ with a coulombic efficiency of 95.27%. After 900 cycles, the discharge capacity was recorded to be 98.8 mAh·g^−1^ with a capacity retention ratio of 89.81% and high coulombic efficiency of >99.5%, demonstrating the feasibility of full cells based on the highly stable UW-Na/Na_3_Zr_2_Si_2_PO_12_ interface. Figure 5b showed electrochemical impedance spectra for the initial and cycled UW-Na | Na_3_Zr_2_Si_2_PO_12_ | Na_3_V_2_(PO_4_)_3_ cell. It was found that the total impedance resistance of the cell shows a slight increase from 400 Ω to 530 Ω. This slight increase of total impedance resistance was mainly ascribed to the increasing of cathode interface resistance because the interface resistance of anode was nearly unchangeable. The typical charge/discharge curves of the assembled UW-Na | Na_3_Zr_2_Si_2_PO_12_ | Na_3_V_2_(PO_4_)_3_ cell at 0.1 mA cm^−2^ was displayed in Figure 5c. During 900 times cycling, the overpotential was is stable at ~150 mV, which also proved the stability of UW-Na/Na_3_Zr_2_Si_2_PO_12_ interface. Fig. 5d showed rate performance of the assembled UW-Na | Na_3_Zr_2_Si_2_PO_12_ | Na_3_V_2_(PO_4_)_3_ cell when charged/discharged with the current densities ranging from 0.05 mA cm^−2^ to 0.5 mA cm^−2^, which delivered reversible capacity of 104.4 mAh·g^−1^ at 0.05 mA cm^−2^, 104.3 mAh·g^−1^ at 0.1 mA cm^−2^, 102.8 mAh·g^−1^ at 0.2 mA cm^−2^, 99.3 mAh·g^−1^ at 0.3 mA cm^−2^ and 93.0 mAh·g^−1^ at 0.5 mA cm^−2^.

The corresponding typical charge/discharge curves in Fig. 5e validate the good rate performance of the cell under different current densities between 2.5–4 V. In addition, the polarization increased from 42 mV to 460 mV can be observed with the increasing of current density from 0.05 mA cm^−2^ to 0.5 mA cm^−2^, which was due to the slow diffusion of sodium ions under the subsequent high current density^46^. The stable interface constructed by ultrasonication between Na and Na_3_Zr_2_Si_2_PO_12_ enables this excellent rate performance^26^. Supplementary Table 2 provides a detail comparison about the reported electrochemical performances of the solid-state sodium metal batteries basing on Na_3_Zr_2_Si_2_PO_12_ electrolyte. UW-Na | Na_3_Zr_2_Si_2_PO_12_ | Na_3_V_2_(PO_4_)_3_ cell assembled by the ultrasound solid welding delivers good cycle stability and competitive rate performance.

In summary, room temperature ultrasonic solid welding strategy was demonstrated in this work to construct intimate contact interface between alkali metal anode and oxide solid-state electrolyte. The intimate contact interface with lower interfacial impedance (~22.6 Ω cm^2^) constructed by ultrasonication enabled the Na symmetric cell could be stably operated at 0.2 mA cm^−2^ for long cycling time at room temperature. Additionally, the significantly higher critical current density (0.6 mA cm^−2^) illustrated the stable plating/stripping behaviour of Na metal during cycling. As a result, the assembled UW-Na | Na_3_Zr_2_Si_2_PO_12_ | Na_3_V_2_(PO_4_)_3_ cell can be well cycled over 900 cycles with a high-capacity retention ratio of 89.81%. This work demonstrated a fast, convenient, low-cost, energy-saving and environmental friendly method to fabricate intimate contact interface between alkali metal anodes and ceramic electrolytes, providing a new gateway to address the interface issues denounced in solid-state metal batteries.

## Methods

### Solid-state electrolyte synthesis

Na_3_Zr_2_Si_2_PO_12_ (NZSP) solid electrolyte was synthesized by the conventional solid-state sintering method. Stoichiometric amount of Na_2_CO_3_ (99.5%, Aladdin), ZrO_2_ (99.99%, Aladdin), SiO_2_ (99.99%, Aladdin) and NH_4_H_2_PO_4_ (99%, Aladdin) raw materials were mixed by ball milling with ZrO_2_ balls (Φ10 mm) at 1200 r/min for 1 h in environment atmosphere, and then calcined at 1100 °C for 12 h in air atmosphere to obtain precursor. 15 wt.% quantities of excessive Na_2_CO_3_ and NH_4_H_2_PO_4_ were added to compensate the volatilization of Na and P elements at high temperature. The obtained precursor was ball-milled at 1200 r/min for 1 h and pressed into green pellets at 200 MPa, then sintered at 1250 °C for 6 h in air atmosphere. The final as-obtained electrolyte pellets were ~800 μm in thickness and ~15 mm in diameter.

### Materials characterizations, cells assemble and electrochemical measurements

The phase structure of the prepared Na_3_Zr_2_Si_2_PO_12_ pellet was analyzed by X-ray diffraction technology (ARL Equinox 3000, France). The microstructure and elemental composition were characterized by field emission scanning electron microscope equipped with energy dispersive spectrometer (Quanta FEG, America). Ionic conductivity of the prepared Na_3_Zr_2_Si_2_PO_12_ pellet was evaluated by the electrochemical impedance spectroscopy (EIS) of the Au | Na_3_Zr_2_Si_2_PO_12_ | Au symmetrical cell operating at a quasi-stationary potential with two-electrode system on an electrochemical workstation (CHI760E, China) in the frequency range from 1 MHz to 1 Hz at open-circuit voltage, 5 mV amplitude. The recording number of data points was 12 (per decade). UW-Na/Na_3_Zr_2_Si_2_PO_12_/Na-UW symmetrical cell was assembled by the proposed ultrasound solid welding for the measurement of interface resistance and cycling stability of the fabricated interface. To evaluate the evolution of interface contact after cycling, the cell was disassembled manually in the glove box by a nipper plier to harvest the solid electrolyte and electrodes for the ex situ SEM measurement transferred by a sealed cans.

The cycle stability of the assembled cells was evaluated by the Galvanostatic cycling measurements at a current density of 0.1~0.2 mA cm^−2^ at room temperature (25–27 °C). The UW-Na | Na_3_Zr_2_Si_2_PO_12_ | Na-UW symmetric cells were tested by rate cycling under the initial current density of 0.1 mA cm^−2^ with an increasing step of 0.05 mA cm^−2^ to determine the critical current density (CCD) at room temperature (25–27 °C). For the assemble of UW-Na | Na_3_Zr_2_Si_2_PO_12_ | Na_3_V_2_(PO_4_)_3_ solid-state sodium metal batteries, Na_3_V_2_(PO_4_)_3_ cathode was prepared by mixing the Na_3_V_2_(PO_4_)_3_/C (Hubei Energy Technology Co., Ltd, China) with acetylene black carbon and PVDF at a weight ratio of 8:1:1 in the solvent of NMP, then the obtained slurry was casted on Al foil and dried at 120 °C in vacuum for 24 h. The load of Na_3_V_2_(PO_4_)_3_/C in the obtained cathode was ~1 mg cm^−2^. The thickness of the Na_3_V_2_(PO_4_)_3_/C-based positive electrode was ~0.033 mm with diameter of 12 mm. CR 2032 coin-type cells were assembled in an argon-filled glovebox ([H_2_O] < 0.1 ppm, [O_2_] < 0.1 ppm) using Na_3_V_2_(PO_4_)_3_/C as the cathode, Na metal as anode and Na_3_Zr_2_Si_2_PO_12_ as electrolyte respectively. The Na anode was ultrasound welding on the one side of the Na_3_Zr_2_Si_2_PO_12_ pellet as described in above. The Na foil (99.7%, Aladdin) was prepared with ~12 mm in diameter and ~120 μm in thickness. 7.5 μL of liquid electrolyte (1 M NaClO_4_ in EC/DMC (1:1) + 5% FEC) was used to wet the interface between cathode and Na_3_Zr_2_Si_2_PO_12_ sheet. The water content of the electrolyte solution ≤50 ppm. The assembled Na metal batteries were galvanostatically charged and discharged in the voltage range of 2.5–4.0 V vs Na/Na^+^ at room temperature (25–27 °C) using an automatic battery tester system (Land CT2001A, China). All the electrochemical measurements were performed on a lab bench without temperature control.

## Supplementary information

Supplementary Information

Description of Additional Supplementary Files

Supplementary Movie 1

## Data Availability

The authors declare that all the relevant data are available within the paper and its Supplementary Information file or from the corresponding author upon reasonable request. Source data are provided with this paper.

## Source data

Source Data
