# Supplementary material for: Improving the alkali metal electrode/inorganic solid electrolyte contact via room-temperature ultrasound solid welding
Source: Nat Commun. 2021 Dec 7;12:7109. doi: 10.1038/s41467-021-27473-4 (PMC8651668; doi:10.1038/s41467-021-27473-4)
Supplement: Supplementary file 1 — Supplementary Information [file 41467_2021_27473_MOESM1_ESM.pdf]

SUPPLEMENTARY INFORMATION

Improving the alkali metal electrode/inorganic solid electrolyte contact via room-temperature ultrasound solid welding

Xinxin Wang[1], Jingjing Chen[2], Dajian Wang[2] & Zhiyong Mao[1]*

[1]Tianjin Key Laboratory for Photoelectric Materials and Devices, School of Materials Science and Engineering, Tianjin University of Technology, Tianjin 300384, PR. China. [2]Key Laboratory of Display Materials and Photoelectric Devices, Tianjin University of Technology, Ministry of Education, Tianjin 300384, PR. China. Correspondence and requests for materials should be addressed to Z. M. (email: mzhy1984@163.com)

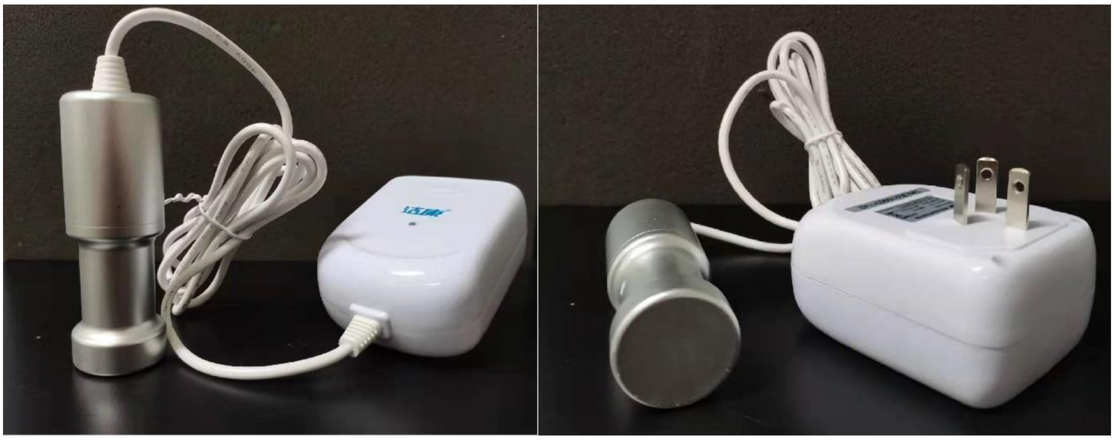

Supplementary Figure 1. Photographic picture of the used household ultrasonic clearer in experiment. JEKEN, CE-9600, power = 70 W, frequency = 50 kHz.

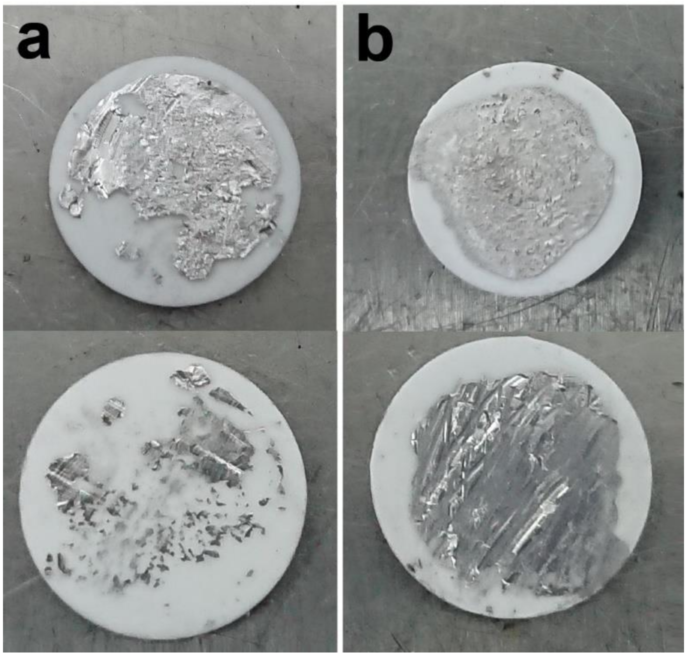

Supplementary Figure 2. Digital photographs for the contact of Na metal foil on the surface of $Na_3Zr_2Si_2PO_{12}$ pellet. **a** Na/$Na_3Zr_2Si_2PO_{12}$. **b** UW-Na/$Na_3Zr_2Si_2PO_{12}$.

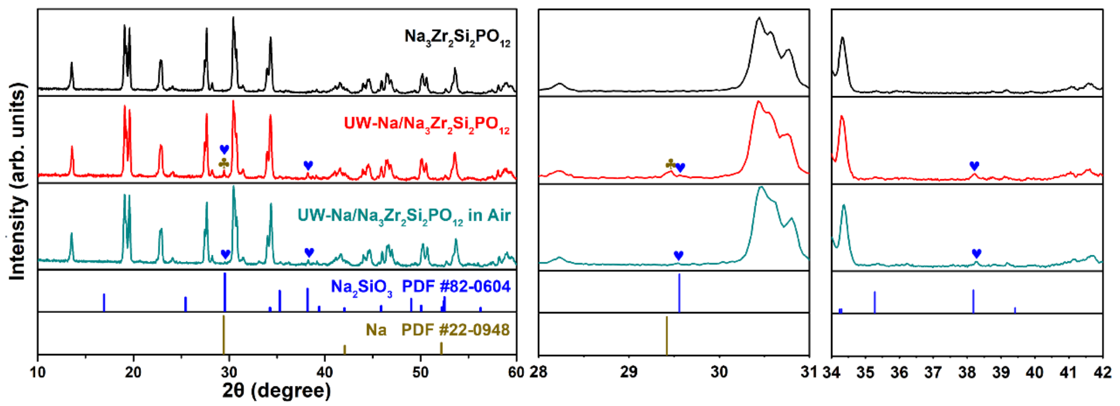

Supplementary Figure 3. XRD diffraction patterns of the ultrasound welding sample of $Na_3Zr_2Si_2PO_{12}$ powder with sodium metal.

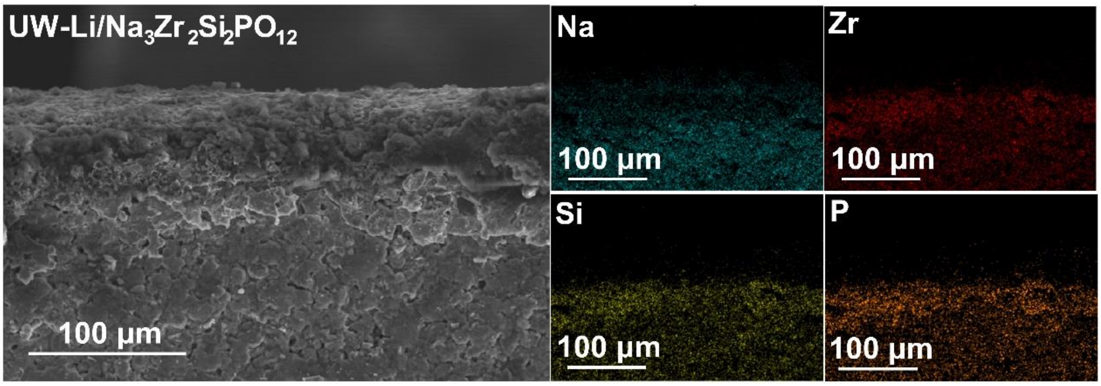

Supplementary Figure 4. SEM image and EDX elemental mappings of the cross-section for UW-Li/$Na_3Zr_2Si_2PO_{12}$ interface.

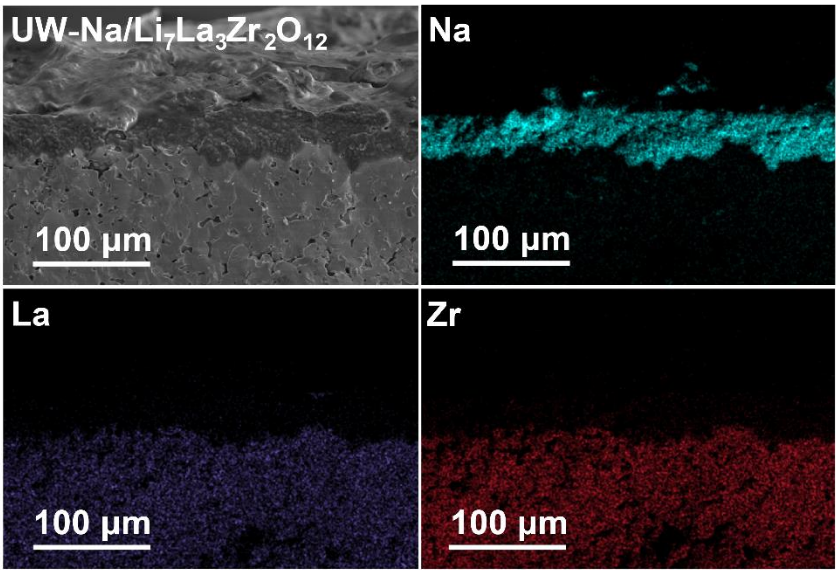

Supplementary Figure 5. SEM image and EDX elemental mappings of the cross-section for UW-Na/$Li_7La_3Zr_2O_{12}$ interface.

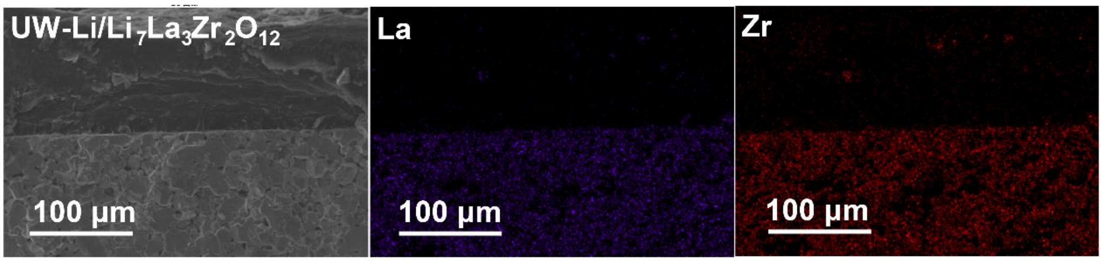

Supplementary Figure 6. SEM image and EDX elemental mappings of the cross-section for UW-Li/$Li_7La_3Zr_2O_{12}$ interface.

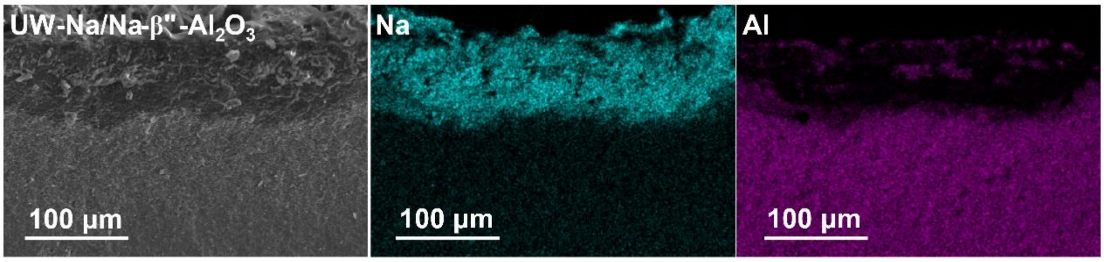

Supplementary Figure 7. SEM image and EDX elemental mappings of the cross-section for UW-Na/Na-β"-Al_2O_3 interface.

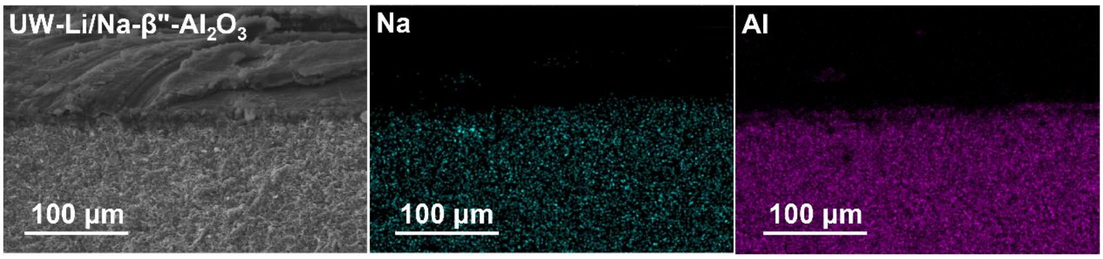

Supplementary Figure 8. SEM image and EDX elemental mappings of the cross-section for UW-Li/Na-β"-Al_2O_3 interface.

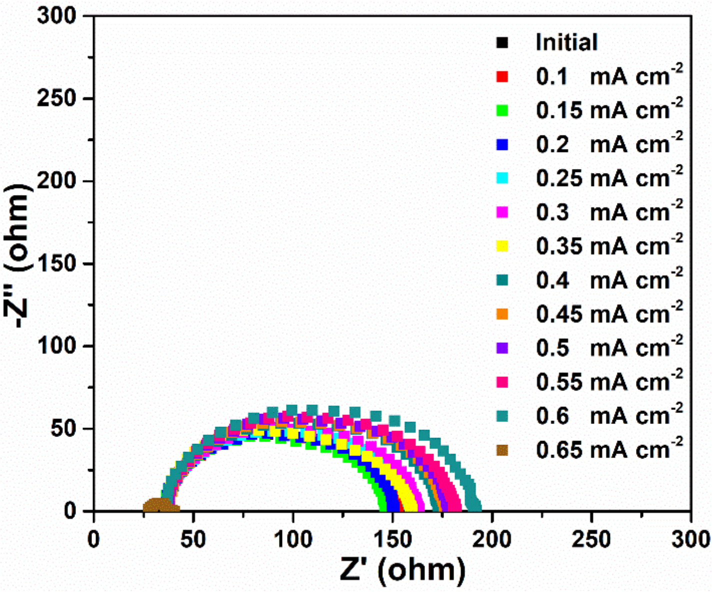

Supplementary Figure 9. EIS measurements of the correlated total electrochemical resistance after the stripping/plating processes of a certain current density (0.1 ~ 0.65 mA cm^{-2}) for UW-Na|$Na_3Zr_2Si_2PO_{12}$|Na-UW cell.

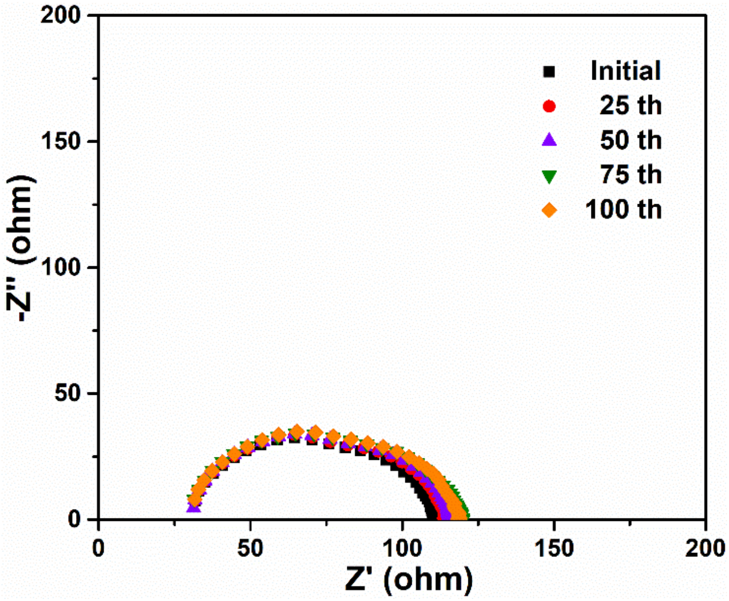

Supplementary Figure 10. The electrochemical impedance spectra for the initial and cycled UW-Na|$Na_3Zr_2Si_2PO_{12}$|Na-UW symmetrical cell at current density of 0.2 mA cm^{-2}.

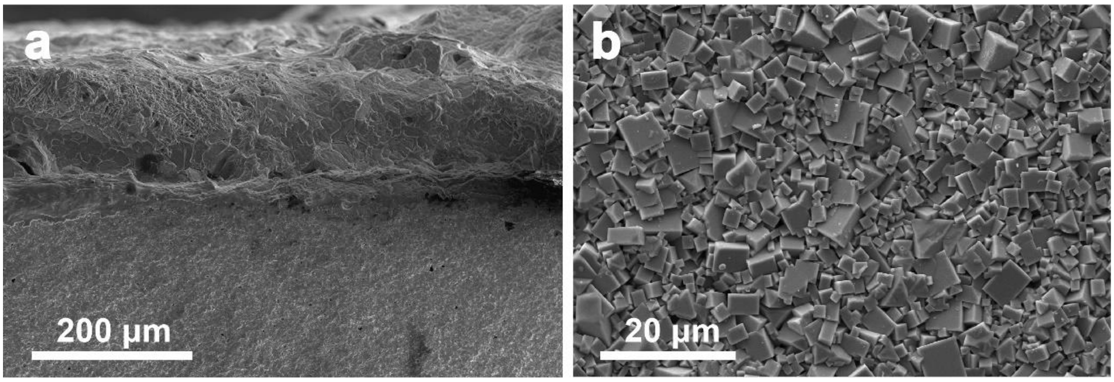

Supplementary Figure 11. SEM image of pellets recovered from cycled UW-Na|$Na_3Zr_2Si_2PO_{12}$|Na-UW cell at 0.2 mA cm^{-2} after 100 h cycling. **a** The cross-section of UW-Na/$Na_3Zr_2Si_2PO_{12}$ interface. **b** Top view of $Na_3Zr_2Si_2PO_{12}$ washed by ethanol.

Supplementary Table 1. Comparison of the interface resistance and cycling stability of $Na|Na_3Zr_2Si_2PO_{12}|Na$ symmetrical cells between recent publications and this work.

Interfacial modification routes	Symmetrical cell impedance	Interface impedance (Ω cm^2)	Critical current density (mA cm^{-2}/ ℃)	Stability (mA cm^{-2}/h/ ℃)	Refs
380 ℃ molten Na/SPS synthesized $Na_3Zr_2Si_2PO_{12}$	400 Ω cm^2	N/A	0.15/65 ℃	0.15/335/65 ℃	1
Na-SiO_2/$Na_3Zr_2Si_2PO_{12}$	575 Ω	101	0.5/RT	0.1-0.2/135/25 ℃	2
Na/AlF_3-$Na_3Zr_2Si_2PO_{12}$	3600 Ω	N/A	1.0/RT, 1.2/60 ℃	0.15-0.25/300/60 ℃	3
Na/TiO_2-$Na_3Zr_2Si_2PO_{12}$	350 Ω cm^2	101	N/A	0.2/750/25 ℃	4
Na/SnO_2-trilayer-0.1Ca-$Na_3Zr_2Si_2PO_{12}$	175 Ω cm^2	N/A	N/A	0.3/400/RT	5
Na/Span-$Na_3Zr_2Si_2PO_{12}$	387.4 Ω cm^2	N/A	1.4/RT	0.15-0.25/500/RT	6
Na/TS-$Na_3Zr_2Si_2PO_{12}$	350 Ω cm^2	129	0.4/RT	0.1/2100/25 ℃<br>0.2/400/25 ℃	7
UW-Na/$Na_3Zr_2Si_2PO_{12}$	140 Ω	22.6	0.6/RT	0.1/1300/RT<br>0.2/400/RT	This work

Supplementary Table 2. Comparison of the electrochemical performances of the solid-state sodium metal cells basing on $Na_3Zr_2Si_2PO_{12}$ solid electrolyte between our work and recent publications.

Battery configuration	Capacity retention ratios	Rate performance	Refs
NVP-LE|$Na_3Zr_2Si_2PO_{12}$-AlF_3|Na	1 C/83.4% after 100 cycles	N/A	3
NVP-LE|$Na_3Zr_2Si_2PO_{12}$-TiO_2|Na	0.1 C/70.6% after 60 cycles	N/A	4
(NVP/SCN/PEO/$NaClO_4$)|0.1Ca-$Na_3Zr_2Si_2PO_{12}$ SnO_2-trilayer|Na	1 C/98.13% after 450 cycles	4 C/80.5 mAh g^{-1}	5
NVP-LE|$Na_3Zr_2Si_2PO_{12}$-SPAN|Na	0.5 C/87.5% after 200 cycles	2 C/81.1 mAh g^{-1}	6
(NVCP/Succinonitrile /$NaClO_4$)|$Na_3Zr_2Si_2PO_{12}$-TS|Na	100 mA g^{-1}/73% after 400 cycles	100 mA g^{-1}/103 mAh g^{-1}	7
NVP-LE|$Na_3Zr_2Si_2PO_{12}$|Na-UW	0.1 mA cm^{-2}/89.81% after 900 cycles	0.5 mA cm^{-2}/93 mAh g^{-1}	This work

Supplementary References:

1. Zhou W, Li Y, Xin S, Goodenough JB. Rechargeable Sodium All-Solid-State Battery. *ACS Cent. Sci.* **3**, 52-57 (2017).
2. Fu H, *et al.* Reducing Interfacial Resistance by Na-SiO_2 Composite Anode for NASICON-Based Solid-State Sodium Battery. *ACS Materials Lett.* **2**, 127-132 (2020).
3. Miao X, *et al.* AlF_3-modified anode-electrolyte interface for effective Na dendrites restriction in NASICON-based solid-state electrolyte. *Energy Stor. Mater.* **30**, 170-178 (2020).
4. Yang JY, *et al.* Guided-formation of a favorable interface for stabilizing Na metal solid-state batteries. *J. Mater. Chem. A* **8**, 7828-7835 (2020).
5. Lu Y, Alonso JA, Yi Q, Lu L, Wang ZL, Sun CW. A High-Performance Monolithic Solid-State Sodium Battery with Ca^{2+} Doped $Na_3Zr_2Si_2PO_{12}$ Electrolyte. *Adv. Energy Mater.* **9**, (2019), 1901205.
6. Miao XG, *et al.* Isotropous Sulfurized Polyacrylonitrile Interlayer with Homogeneous Na+ Flux Dynamics for Solid-State Na Metal Batteries. *Adv. Energy Mater.* **11**, (2021), 202003469.
7. Wang CZ, Jin HB, Zhao YJ. Surface Potential Regulation Realizing Stable Sodium/$Na_3Zr_2Si_2PO_{12}$ Interface for Room-Temperature Sodium Metal Batteries. *Small* **17**, (2021), 202100974.
