# Supplementary material for: Improving the alkali metal electrode/inorganic solid electrolyte contact via room-temperature ultrasound solid welding
Source: Nat Commun. 2021 Dec 7;12:7109. doi: 10.1038/s41467-021-27473-4 (PMC8651668; doi:10.1038/s41467-021-27473-4)
Supplement: Supplementary file 2 — Description of Additional Supplementary Files [file 41467_2021_27473_MOESM2_ESM.pdf]

Description of additional Supplementary data files

Supplementary Movie: Video of the ultrasound welding procedure
